# Radiomics and artificial intelligence in precision radiotherapy for cervical cancer: a narrative review

**DOI:** 10.3389/fonc.2026.1781422

**Published:** 2026-04-07

**Authors:** Yuxin He, Yuzhou Lu, Lijun Hu

**Affiliations:** Department of Radiation Oncology, Changzhou Second People’s Hospital, Nanjing Medical University, Changzhou, China

**Keywords:** artificial intelligence, cervical cancer, prognosis, radiomics, radiotherapy

## Abstract

Cervical cancer (CC) continues to impose a substantial global health burden and remains one of the most prevalent malignancies among women worldwide. Radiotherapy is a cornerstone treatment for locally advanced disease, and its precision critically impacts tumor control and treatment-related toxicity. Within the evolving paradigm of precision oncology, radiomics and artificial intelligence (AI) have emerged as promising tools to personalize radiotherapy by improving target delineation, predicting treatment response, refining prognostic stratification, and facilitating individualized toxicity risk assessment. This narrative review synthesizes and critically appraises the current evidence on the application of radiomics and AI in CC radiotherapy, focusing on three principal domains: automated target volume delineation, prediction of prognosis and treatment response, and forecasting of radiotherapy-induced toxicities. We further evaluate the methodological rigor and translational readiness of existing studies. Despite encouraging technical performance, most available evidence remains retrospective, with limited prospective validation and uncertain impact on clinical decision-making. Clinical implementation is further challenged by imaging heterogeneity, insufficient standardization, and limited model interpretability. Future research should prioritize large-scale multicenter validation, methodological standardization, and prospective evaluation to determine whether radiomics-guided strategies can meaningfully improve patient outcomes and support integration into routine clinical practice.

## 1 Introduction

Cervical cancer (CC) remains the fourth most commonly diagnosed malignancy among women worldwide. According to GLOBOCAN 2022 estimates, approximately 660,000 new cases and 350,000 deaths occur annually (1). Most patients present with locally advanced cervical cancer (LACC), for which radiotherapy represents a cornerstone curative modality (2). Concurrent chemoradiotherapy (CCRT) is the established standard of care for LACC and has significantly improved local control and overall survival (OS). Despite technological advances from 2D radiotherapy to modern intensity-modulated radiotherapy and image-guided radiotherapy, which have markedly enhanced dose conformity and delivery accuracy, clinical outcomes remain heterogeneous across individuals.

Furthermore, treatment-related toxicities such as bone marrow suppression, gastrointestinal reactions, and radiation-induced proctitis remain prevalent (3). Approximately 35% of patients experience treatment resistance, leading to local failure, recurrence, or distant metastasis (4). Consequently, early prediction of therapeutic response and prognosis is essential to facilitate individualized treatment strategies, thereby optimizing clinical benefits while mitigating unnecessary toxicities and the associated socioeconomic burden.

**Figure 1:**
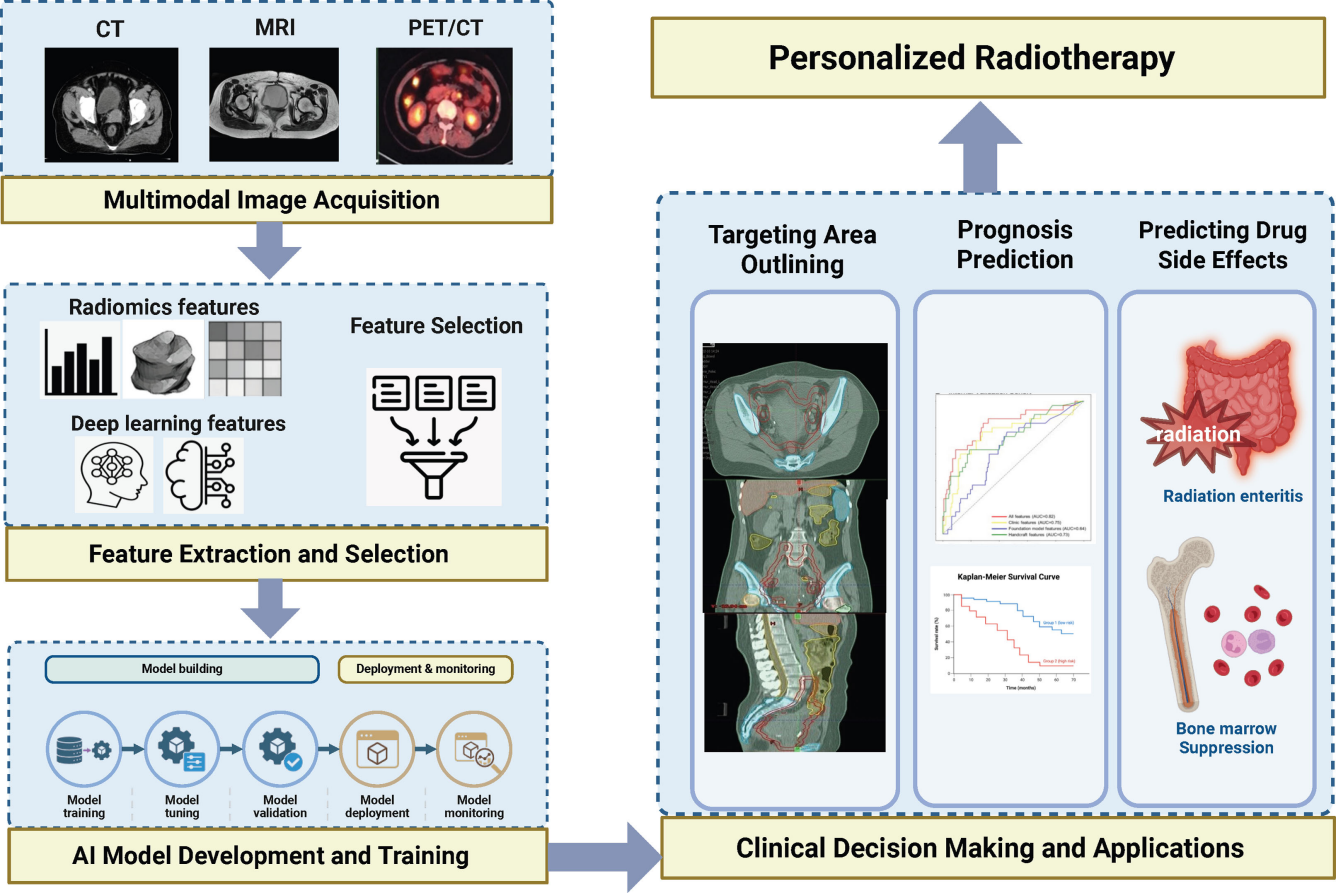
An integrated workflow of artificial intelligence and radiomics for personalized radiotherapy in cervical cancer.

**Figure 2:**
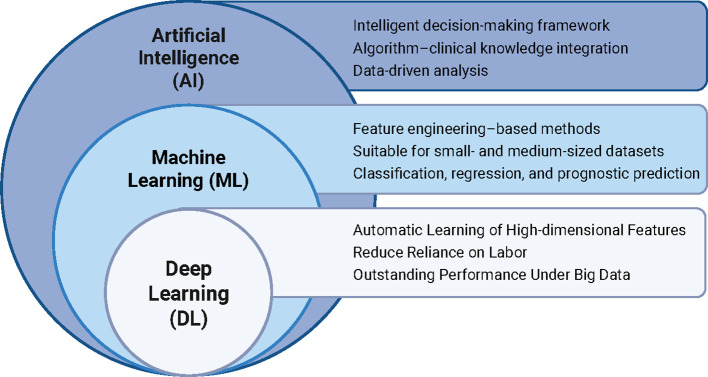
The hierarchy of AI, ML, and DL in radiomics research.

Radiomics and artificial intelligence (AI) offer promising approaches to address these challenges (5, 6). Radiomics facilitates quantitative characterization of intratumoral heterogeneity through high-throughput extraction of imaging features derived from standardized medical imaging (7–10). AI-driven modeling further enables the integration of high-dimensional imaging data with clinical variables, uncovering complex associations relevant to treatment response and survival outcomes. Since its introduction in 2012 (11), radiomics has increasingly been applied in CC management, particularly in target volume delineation, treatment response prediction, and toxicity assessment (**Figure 1**) (12–19). Machine learning (ML) and deep learning (DL) constitute the principal methodological frameworks underpinning radiomics research (**Figure 2**) (20, 21). ML algorithms construct predictive models based on engineered features, such as texture, shape, and intensity statistics, and are particularly suitable for datasets with limited sample sizes (9). In contrast, DL employs multilayer neural networks, including convolutional neural networks (CNNs) and vision transformers, to automatically learn hierarchical representations directly from imaging data (22, 23). By reducing reliance on handcrafted features, DL has demonstrated superior performance in pattern recognition and predictive modeling, particularly when supported by large-scale datasets (24).

Nevertheless, the clinical translatability of current research remains hampered by significant methodological limitations, including inadequate data standardization, overfitting in small cohorts, and a lack of robust external validation (14, 25–27). Further investigation into model interpretability and robust validation strategies is warranted to facilitate clinical translation.

This narrative review synthesizes current evidence regarding radiomics and AI in CC radiotherapy across three key domains: automated target volume delineation, treatment response assessment, and toxicity prediction. In addition to summarizing technical advancements, we discuss the methodological characteristics and the current readiness for clinical implementation of these approaches. By evaluating the balance between technical performance and clinical utility, this review aims to provide perspectives on the requirements for advancing precision and personalized radiotherapy in CC.

## 2 Structured translational appraisal framework

To provide a structured overview of the heterogeneous literature, we developed a qualitative framework inspired by the radiomics lifecycle and readiness concepts proposed by Lambin et al. (28, 29). Rather than applying a formal scoring tool, this framework serves as a descriptive lens to contextualize methodological maturity and translational progress. This review is narrative in nature and does not aim to provide systematic evidence synthesis or formal risk-of-bias scoring.

### 2.1 Appraisal dimensions

The appraisal framework focuses on two complementary dimensions:

#### 2.1.1 Translational validation and clinical integration

Studies were descriptively categorized according to their validation strategy and engagement with clinical applicability:

- Exploratory stage: Retrospective, single-center studies relying solely on internal validation and primarily reporting technical performance metrics without addressing workflow integration or clinical decision impact.
- Early validation stage: Studies incorporating resampling methods, limited multicenter data, or independent external cohorts, and acknowledging potential clinical application, but without prospective evaluation of decision impact.
- Advanced validation stage: Studies including robust independent external validation or multicenter designs with explicit discussion of workflow feasibility and clinical implications. Notably, no study has yet demonstrated prospective patient-level benefit.

These categories reflect relative translational progress rather than formal quality rankings.

#### 2.1.2 Overfitting risk

The potential risk of overfitting was qualitatively appraised by considering cohort size, feature dimensionality, and the rigor of the validation strategy. Particular attention was paid to the relationship between feature number and sample size, the application of regularization approaches, the use of internal resampling techniques, and whether independent external validation was performed. Based on these considerations, studies were broadly interpreted as having a relatively low, moderate, or high susceptibility to overfitting.

### 2.2 Application of the framework

The framework was applied descriptively across the included studies to facilitate structured comparison. Predictive performance and translational appraisal are summarized separately. **Tables 1**, **2** provide a technical overview of model characteristics and reported performance metrics, whereas **Tables 3**–**5** summarize study design rigor and translational maturity. Because statistical accuracy does not necessarily imply clinical readiness, performance, and methodological appraisal are presented separately to support balanced interpretation.

**Table 1:** A summary of studies applying radiomics to predict survival outcomes in radiotherapy for cervical cancer.

Studies	Survival outcome	Region	Feature types	Algorithm	Total cases	Evaluation metrics
Lin et al. (67)	OS	Primary tumor site	Clinical; MRI radiomics	Radiomics nomogram (Radio + Clinical)	582	Nomogram AUC = 0.862 (Test)
Shi et al. (68)	OS	Primary tumor site	PET/CT radiomics	Radiomics model (Cox; LASSO-Cox; RSF)	154	C-index of Cox; LASSO-Cox; RSF = 0.691 ± 0.026; 0.634 ± 0.018; 0.684 ± 0.020
Florit et al. (12)	DFS	Primary tumor site	Clinical; Baseline; Delta PET/CT radiomics	Radiomics model (RSF)	95	C-index ≤ 0.72
	OS					C-index of radiomic; combined model = 0.79
Kari et al. (69)	PFS	Primary tumor site	Pre-treatment T2WI radiomics; Delta T2WI radiomics	Radiomics model (LASSO-Cox)	110	Pre-treatment-rad yielded AUC = 0.75; Delta-rad yielded AUC = 0.79
Wang et al. (70)	DFS	Primary tumor site	Pre-treatment CT radiomics; Pre-treatment CT deep features; Post-treatment radiomics; Post-treatment deep features	CerviPro model	1018	AUC (internal validation) = 0.79-0.80; AUC (external validation) = 0.63-0.64
Park et al. (71)	OS	Primary tumor; Involved lymph node	Radiomics	Radiomics model (RSF)	93	AUC (Validation) = 0.634-0.796
Li et al. (72)	PFS	Spleen	Clinical; Pre-treatment CT radiomics; Post-treatment CT radiomics	Radiomics model (Cox regression)	257	AUC (Validation) = 0.895

OS, Overall Survival; IVIM, Intravoxel Incoherent Motion; DWI, Diffusion Weighted Imaging; MRI, Magnetic Resonance Imaging; AUC, Area Under the Curve; DFS, Disease-Free Survival; PET/CT, Positron Emission Tomography/Computed Tomography; LASSO, Least Absolute Shrinkage and Selection Operator; Cox, Cox regression; RSF, Random Survival Forest; C-index, Concordance index; PFS, Progression-Free Survival; T2WI, T2-weighted Imaging; CT, Computed Tomography.

**Table 2:** A Summary of studies applying radiomics to predict toxic side effects in radiotherapy for cervical cancer.

Studies	Toxic effect	Images	Region	Feature types	Algorithm	Total cases	Evaluation metrics
Zhang et al. (82)	Radiation proctitis	CT	Rectum	Clinical; Pre-CCRT CT radiomics	SAM-Med2D DL model	150	AUC (LR) = 0.73 AUC (RF) = 0.69 AUC (GNB) = 0.63
Lucia et al. (83)	Rectal, GU, and vaginal toxicities	3D Dose	Rectum; GU; Vaginal	Clinical; DVH; 3D dose parameters; Radiomics	NTCP model (unimodal features); NTCP model (multimodal combinations)	102	AUC (Rectal Acute; Test) = 0.76; AUC (GU Acute; Test) = 0.83; AUC (Vaginal Acute; Test) = 0.82; AUC (Rectal Late; Test) = 0.76; AUC (GU Late; Test) = 0.83; AUC (Vaginal Late; Test) = 0.82
Xue et al. (84)	Radiation proctitis	MRI	Rectum	Clinical; Radiomics	Delta radiomics model (RF)	126	AUC (Test) = 0.90
Wei et al. (85)	Radiation proctitis	CT	Rectum	Clinical; Dose-volume parameters; Radiomics	Radiomics model (MLR)	178	AUC (Test) = 0.685
Luo et al. (86)	HT	CT; MRI	Pelvic bone marrow	Clinical; Radiomics	Radiomics model (LR; RF; SVM; XGBoost; NN)	534	AUC (XGBoost; Test) = 0.927; AUC (NN; Test) = 0.891; AUC (RF; Test) = 0.929; AUC (SVM; Test) = 0.917
Yue et al. (87)	HT	CT	Bone marrow; Femoral head; CTV	Demographic; Clinical; Dosimetric; Radiomics	SVM-RFE	683	BM AUC = 0.737 (Radiotherapy-alone; Validation) BM AUC = 0.802 (CCRT; Validation) BM AUC = 0.793 (Combined; Validation)
Tang et al. (88)	Bone Marrow Suppression	CT	Pelvic bone marrow	Clinical; Dosimetric; Radiomics	RF model	106	AUC = 0.743 (Combined Model; Test)
Wang et al. (89)	HT	MRI (IDEAL IQ)	Pelvic bone marrow	PDFF%	Spearman correlation	54	ANC nadir at 5–10 Gy, r = 0.62; P = 0.006 ALC nadir at 5–10 Gy, r = 0.554, P = 0.017
Qin et al. (90)	HT	MRI (IDEAL IQ & T2 fat suppression sequences)	Pelvic bone marrow	Radiomics	Pearson correlation	54	IDEAL IQ (firstorder-Range) vs ΔANC-END at 5–10 Gy, r = 0.744; p = 0.001; T2fs (firstorder-10Percentile) vs ΔANC-END at 5–10 Gy, r = -0.654; p = 0.004; T2fs (firstorder-10Percentile) vs ΔWBC-END at 5–10 Gy, r = -0.563; p = 0.019

CT, Computed Tomography; SAM-Med2D, Segment Anything Model for Medical 2D; DL, Deep Learning; AUC, Area Under the Curve; LR, Logistic Regression; RF, Random Forest; GNB, Gaussian Naive Bayes; GU, Genitourinary; NTCP, Normal Tissue Complication Probability; MRI, Magnetic Resonance Imaging; MLR, Multivariate logistic regression; HT, Hematologic Toxicity; SVM, Support Vector Machine; XGBoost, eXtreme Gradient Boosting; NN, Neural Network; SVM-RFE, Support Vector Machine-Recursive Feature Elimination; BM, Bone Marrow; CTV, Clinical Target Volume; CCRT, Concurrent Chemoradiotherapy; IDEAL IQ, an MRI sequence (Iterative Decomposition of water and fat with Echo Asymmetry and Least-squares estimation) that quantifies fat and water content separately; PDFF%, Proton Density Fat Fraction; ANC, Absolute Neutrophil Count; ALC, Absolute Lymphocyte Count; Nadir, In oncology, this refers to the lowest value (e.g., cell count) reached by a laboratory parameter during a course of therapy, often used to grade the severity of treatment-related toxicity; WBC, White Blood Cell; First-order Radiomics Features, describe the distribution of voxel intensities within an image region (e.g., Range, 10Percentile), without considering spatial relationships; ΔANC-END/ΔWBC-END, the rate of change in ANC/WBC counts from before treatment to the end of radiotherapy.

**Table 3:** Translational evaluation of AI-driven target delineation and workflow optimization in radiotherapy.

Studies	Study aim	Study design	Sample size	Model development and validation framework	Reported performance	Translational stage	Major limitations
Xu et al. (41)	Auto-segmentation (CTV + PTV)	Retrospective (multicenter)	602	5-fold CV + augmentation + hyperparameter tuning; external validation.	DSCs: 83.42%	Validation study	Performance drop in the external cohort; indistinct CTV boundaries; no clinical impact analysis.
Rouhi et al. (42)	Auto-segmentation (GTV)	Retrospective and prospective external validation	166	5-fold CV + ensemble learning + SMOTE + nested CV + prospective external validation.	DSCs: 72.00%	Validation study	2D MRI only; image quality variability; no dosimetric/clinical validation.
Yoganathan et al. (43)	Auto-segmentation (GTV + CTV)	Retrospective (single-center)	39 (71 datasets)	augmentation + weighted loss + regularization; no CV; no external validation.	DSCs: 62.00%	Exploratory study	Very small cohort; high overfitting risk; no external validation.
Shi et al. (44)	Auto-segmentation (CTV)	Retrospective (multicenter)	621	Two-stage training + augmentation + weight decay; external validation.	DSCs: 81.33%	Validation study	Small external cohort; no prospective validation.
Gu et al. (45)	Auto-segmentation (OARs)	Retrospective (single-center)	95	5-fold CV + augmentation; no external validation.	DSCs: 73.69%	Validation study	Small cohort; poor performance for small structures; no external or clinical validation.
Wang et al. (37)	Auto-contouring enhancement (LLM)	Retrospective (single-center)	32 patients; 124 CT images	4-fold CV + augmentation + dynamic routing modules; no external validation.	DSCs: 78.00–91.00%	Exploratory study	Small cohort; no external validation.
Liang et al. (53)	External beam auto-planning	Retrospective (multicenter)	221	5-fold CV + held-out test set + regularization and early stopping; no external validation.	MAE <5%; CI 0.915–0.921	Validation study	No external validation; adaptation across planning styles.
Sun et al. (54)	AI-driven oART framework	Retrospective (multicenter)	24 patients; 671 fractions	5-fold CV with class balancing+ augmentation + dropout; no independent external validation.	AUC 0.906–0.917	Exploratory study	Very small patient cohort; class imbalance; no outcome validation.
Mason et al. (55)	Auto-plan selection (POTD)	Retrospective (single-center)	39 patients; 226 CBCTs	Strict data split + augmentation + patient-specific prior; no external validation.	DSCs: 78.00–94.00%	Exploratory study	Limited rare anatomies; no external or prospective validation.

CTV, Clinical Target Volume; PTV, Planning Target Volume; CV, Cross-Validation; DSCs, Dice Similarity Coefficient; GTV, Gross Tumor Volume; SMOTE, Synthetic Minority Over-sampling Technique; MRI, Magnetic Resonance Imaging; OARs, Organs at Risk; LLM, Large Language Model; CT, Computed Tomography; MAE, Mean Absolute Error; CI, Conformity Index; AI, Artificial Intelligence; oART, online adaptive radiotherapy; AUC, Area Under the Curve; POTD, plan-of-the-day; CBCT, Cone-Beam Computed Tomography.

**Table 4:** Methodological quality and clinical readiness of AI-based predictive models in radiotherapy.

Studies	Study design	Sample size	Methodological framework	Overfitting risk	Clinical readiness stage	Major limitations
Gui et al. (60)	Retrospective dual-center pooled	183	ML comparison with internal optimization; no external validation.	High	Exploratory	No independent validation; limited model interpretability.
Liu et al. (61)	Retrospective (single-center)	164	Multivariable model with internal thresholding; no external validation.	Moderate	Exploratory	No external validation; lack of resampling; limited generalizability.
Zhang et al. (62)	Retrospective (single-center)	163	LASSO-based model with internal split validation; no external validation.	Moderate	Early validation stage.	No external validation; short-term outcome definition.
Watanabe et al. (63)	Retrospective (single-center)	103	Cox regression with internal ROC validation; no external validation.	Moderate	Exploratory	No external validation; heterogeneous treatment exposure.
Collarino et al. (64)	Retrospective (single-center)	195	Feature reduction only; no final predictive model.	N/A	Exploratory	No final predictive model developed; all AUCs <0.70; limited biological interpretability.
Lin et al. (67)	Retrospective (multicenter)	582	LASSO-based radiomics score with multivariable Cox modeling; multicenter external validation.	Moderate	Early validation stage	No prospective validation; no formal fairness assessment; no cost-effectiveness analysis.
Shi et al. (68)	Retrospective (single-center)	154	Unsupervised clustering with multivariable modeling; internal split + repeated 3-fold CV; no external validation.	Moderate	Exploratory	No independent external validation; moderate predictive performance; potential model instability.
Florit et al. (12)	Retrospective (single-center)	95	Radiomic modeling with stratified 5-fold CV; no external validation.	Moderate	Exploratory	No external validation; small cohort; poor calibration.
Wagner-Larsen et al. (69)	Retrospective (multicenter)	110	LASSO Cox with internal training/validation split; no external validation.	Moderate	Early validation stage	Small cohort; internal validation only; segmentation variability; scanner heterogeneity.
Wang et al. (70)	Retrospective (multicenter)	1018	Multimodal DL with internal + external validation.	Moderate	Early validation stage	Reduced performance in external cohorts; no prospective validation.
Park et al. (71)	Retrospective (single-center)	93	ML (RSF), feature clustering, internal validation only; no external validation.	High	Exploratory	No external validation; limited interpretability.
Li et al. (72)	Retrospective (single-center)	257	LASSO Cox, radiomics + clinical model, internal validation, nomogram; no external validation.	Moderate	Exploratory	No external validation; retrospective design.
Dankulchai et al. (75)	Retrospective (single-center)	90	Feature selection + Cox; no validation cohort.	High	Exploratory	Small sample; no independent validation; high overfitting risk.
Kawahara et al. (76)	Retrospective (single-center)	89	LASSO feature selection + neural network; internal split with 5-fold CV; no external validation.	High	Exploratory	Small sample; no external validation; limited interpretability.
Yusufaly et al. (77)	Retrospective (single-center)	127	Bagging-based feature screening + Cox model; internal split with bootstrap validation; no external validation.	Moderate	Exploratory	Limited events; no external validation; limited generalizability.
Mu et al. (78)	Retrospective (multicenter)	154	LASSO-based radiomics score with multivariable Cox modeling; internal CV and multicenter external validation.	Moderate	Early validation stage	No prospective validation; limited explainability; moderate sample size.

ML, Machine Learning; LASSO, Least Absolute Shrinkage and Selection Operator; Cox, Cox regression; ROC, Receiver Operating Characteristic; N/A, Not Applicable; AUC, Area Under the Curve; CV, Cross-Validation; DL, Deep Learning; RSF, Random Survival Forest.

**Table 5:** Methodological quality and clinical readiness of AI-based predictive models in radiotherapy.

Studies	Design risk	Sample size	Methodological framework	Overfitting risk	Clinical readiness stage	Major limitations
Zhang et al. (82)	Retrospective (single-center)	120	DL feature extraction with LASSO selection; ML comparison using 5-fold CV; no external validation.	High	Exploratory	No external validation; small sample; limited clinical integration; no prospective design.
Lucia et al. (83)	Retrospective (multicenter)	102	Radiomics-based logistic modeling with feature reduction; internal split and multicenter external validation.	Moderate	Early validation stage	Small cohort; no prospective validation; limited fairness analysis.
Xue et al. (84)	Retrospective (single-center)	126	ML feature selection (LR + LASSO + RF); internal split validation only; no external validation.	High	Exploratory	No external validation; small sample; SMOTE use; no prospective design.
Wei et al. (85)	Retrospective (single-center)	178	LASSO + multivariate logistic regression; no external validation.	Moderate	Exploratory	No external validation; modest AUC; limited generalizability.
Luo et al. (86)	Retrospective (single-center)	534	Multi-ML comparison; multimodal integration; no external validation.	Moderate	Early validation stage	No external multicenter validation; no prospective validation; no fairness analysis.
Yue et al. (87)	Retrospective (single-center)	683	Radiomics + dosimetric + clinical integration; SVM-RFE feature selection; multi-ML modeling; external validation.	Moderate	Early validation stage	No prospective validation; potential SMOTE bias; limited fairness and real-world evaluation.
Tang et al. (88)	Retrospective (single-center)	106	LASSO feature selection; ML comparison using cross-validation; no external validation.	High	Exploratory	No external validation; small sample; limited clinical integration.
Wang et al. (89)	Prospective observational study (single-center)	54	Quantitative MRI dose-gradient analysis; correlation modeling; no predictive model.	N/A	Exploratory	Small sample; mechanistic rather than predictive modeling; limited clinical decision integration.
Qin et al. (90)	Prospective (multicenter)	54	Multi-sequence MRI radiomics extraction; statistical feature reduction; dose-response and correlation analysis; no predictive model.	N/A	Exploratory	Small sample; correlation-based analysis rather than predictive modeling; limited translational applicability.

DL, Deep Learning; LR, Logistic Regression; LASSO, Least Absolute Shrinkage and Selection Operator; RF, Random Forest; ML, Machine Learning; CV, Cross-Validation; SMOTE, Synthetic Minority Over-sampling Technique; AUC, Area Under the Curve; SVM-RFE, Support Vector Machine - Recursive Feature Elimination; N/A, Not Applicable; MRI, Magnetic Resonance Imaging.

## 3 Core applications of radiomics in radiotherapy for cervical cancer

### 3.1 The role of radiomics in target delineation for cervical cancer radiotherapy

#### 3.1.1 Clinical challenges and limitations of target area delineation

Accurate delineation of the clinical target volume (CTV) and organs at risk (OARs) is essential for effective radiotherapy in CC. However, the CTV often lacks well-defined radiographic boundaries and remains highly dependent on physician expertise, resulting in subjectivity, inter-observer variability, and limited reproducibility. In addition, manual delineation is labor-intensive, typically requiring 20–40 minutes per case (30, 31).

Critical OARs, including the rectum and sigmoid colon, are anatomically adjacent to the target volume and are frequently encompassed within the delineation field. These structures are characterized by low soft-tissue contrast and complex anatomical variability, increasing the risk of inaccuracies with conventional manual approaches (32). To address these limitations, atlas-based and graph-based automated segmentation techniques have been developed (33–35). Compared with manual delineation, automated methods substantially reduce contouring time, improve consistency, and decrease inter-operator variability, particularly in anatomically stable regions with well-defined boundaries.

Nevertheless, significant challenges remain in CC applications. The performance of atlas-based methods is constrained by atlas quality and dataset representativeness, and segmentation accuracy remains suboptimal in regions with marked anatomical variation, such as the mesenteric recess (36).

#### 3.1.2 Artificial intelligence–driven automated target segmentation

DL models, predominantly based on CNNs and Transformer architectures, have been increasingly applied to automated target segmentation using computed tomography (CT) and magnetic resonance imaging (MRI) (37–40). Several studies have reported promising segmentation performance across diverse anatomical targets.

Xu et al. (41) developed a multicenter DL model for automatic CTV and planning target volume (PTV) segmentation in uterine malignancies, achieving mean Dice similarity coefficients (DSCs) of 83.42% for PTV and 81.23% for CTV. Notably, approximately 90% of contours generated in the external validation cohort required only minor manual refinement, indicating reasonable cross-institutional generalizability. Rouhi et al. (42) compared eight DL architectures for gross tumor volume segmentation and demonstrated that 2D networks outperformed 3D models when applied to 2D MRI sequences. They further proposed a radiomics-based failure detection framework to enhance model reliability. Yoganathan et al. (43) introduced an anatomical dual-prior network that explicitly incorporated spatial relationships between the psoas muscle and the CTV. Their 2.5D architecture achieved superior performance compared with conventional 2D approaches. Similarly, Shi et al. (44) developed a dual-prior model trained on 621 patients, reporting a DSC of 81.33%, exceeding that of standard 3D U-Net architectures. The relatively large sample size and multicenter design strengthen the methodological rigor of this study; however, prospective validation and clinical implementation in routine practice remain to be further explored. MFFUNet, a hybrid convolutional neural network–Transformer architecture, integrates CNN-based local feature extraction with Transformer-based global context modeling via a cross-attention fusion module, achieving a DSC of 73.69% for OAR segmentation (45). In addition to improving segmentation accuracy, this framework reduced contouring time to less than one minute per case.

Despite these advances, most studies focus primarily on technical performance metrics, such as DSC, with limited evaluation of their dosimetric impact or implications for clinical decision-making. Additionally, while several multicenter studies with external validation have been reported, most remain retrospective and lack prospective integration into clinical workflows, with insufficient attention given to clinician involvement in the validation process.

#### 3.1.3 Automated dose protection and segmentation for critical organs

Accurate delineation of tumor targets is fundamental to CC radiotherapy; however, optimal protection of OARs is equally essential for minimizing radiation-induced toxicity (46, 47). AI-driven segmentation models have demonstrated favorable performance for relatively well-defined pelvic OARs, such as the bladder and rectum. In contrast, anatomically complex and highly variable structures such as the sigmoid colon and small bowel loops remain challenging (45).

Recent studies integrating large language models with DL architectures have reported improved segmentation consistency across selected OARs (37). DSCs exceeding 0.90 have been achieved for bladder segmentation, whereas performance for the rectum is moderate and substantially lower for the sigmoid colon. These discrepancies highlight persistent challenges in modeling irregular anatomical boundaries and inter-patient anatomical variability. Although some investigations incorporate multicenter datasets, independent external validation remains inconsistent, and performance stability across heterogeneous imaging protocols and scanner platforms has not been systematically established.

OARs protection strategies may be further enhanced by integrating radiomic features with dose–volume histogram (DVH) parameters. For example, pelvic bone marrow dose metrics (V5, V20, and V30, representing the percentage volume receiving at least 5, 20, and 30 Gy) have been associated with acute hematologic toxicity (45). However, DVH-derived parameters provide limited spatial information and do not capture the 3D heterogeneity of dose distribution, thereby constraining the robustness of toxicity prediction models (48). Moreover, validation cohorts in many studies share similar imaging characteristics and acquisition protocols, limiting confidence in generalizability across heterogeneous clinical settings and institutions.

Despite these technical advances, important methodological limitations restrict translational readiness. According to the Structured Translational Appraisal Framework (validation robustness, overfitting risk, and clinical readiness), most studies remain retrospective, are based on single-institution cohorts of modest size, and lack rigorous independent external validation. Even when external datasets are used, the variation in anatomical presentation and imaging techniques is still not fully addressed, which undermines confidence in the consistency of structural results (45). In addition, models incorporating high-dimensional radiomic features remain susceptible to overfitting, while systematic mitigation strategies are inconsistently reported.

Overall, although AI-assisted OAR segmentation and dose-protection modeling demonstrate promising technical performance, the current evidence largely remains within exploratory to early validation stages. Multicenter robustness, prospective validation, and clear demonstration of clinical benefit are required before routine implementation in clinical practice can be justified.

#### 3.1.4 Artificial intelligence developments for radiotherapy process optimization

Building upon automated segmentation, AI has progressively expanded into downstream radiotherapy workflow optimization, particularly in automated treatment planning and adaptive replanning. These systems aim to address limitations of conventional knowledge-based planning by enabling dynamic optimization under complex constraints and evolving anatomical conditions (49–51). In adaptive radiotherapy, AI-driven models facilitate rapid replanning following repeat imaging, thereby enabling more individualized dose delivery throughout the treatment course (52).

To address data imbalance in CT-based knowledge-based planning, Liang et al. (53) compared three calibration strategies for dose prediction, including prediction tolerance adjustment, transfer learning, and hybrid density networks. All approaches generated clinically acceptable plans with reduced OAR doses. Hybrid density networks demonstrated the most consistent predictive performance, whereas transfer learning required less training data. In the setting of online adaptive radiotherapy for CC, Sun et al. (54) developed ML and DL-based decision models using 671 treatment fractions. Among the evaluated approaches, a contour-only DL model achieved the highest performance, with an accuracy of 0.869 and an area under the curve (AUC) of 0.917, and showed greater agreement with reference standards than physician consensus. Mason et al. (55) introduced the U-Seg3 (automated segmentation system name) system for automated segmentation on cone beam CT. When combined with predefined quantitative criteria, the system achieved a plan selection rate of 77%, comparable to that of experienced radiation therapists.

Collectively, these studies indicate a gradual transition from AI-assisted target delineation toward integrated delineation and treatment planning workflows (56, 57). However, the current evidence remains preliminary. As summarized in **Table 3**, according to the Structured Translational Appraisal Framework, most workflow optimization studies are retrospective, conducted at single institutions, and based on limited validation strategies. Outcomes are primarily measured by agreement with expert assessment rather than prospective clinical endpoints. Although performance metrics such as accuracy, AUC, and plan selection rate are frequently reported, it remains unclear whether these improvements translate into reductions in toxicity, improvements in workflow efficiency, enhanced resource utilization, or survival benefit.

Moreover, cross-institutional validation under heterogeneous imaging conditions and adaptive workflows is rarely performed, limiting confidence in generalizability. Practical implementation factors are also insufficiently examined, including integration into existing planning systems, computational efficiency, regulatory considerations, and clinician acceptance. Therefore, while technical feasibility has been demonstrated, current evidence does not support routine clinical implementation. Future investigations should prioritize prospective multicenter validation, standardized performance benchmarks, transparent reporting of workflow efficiency, and demonstration of patient-centered clinical benefit before widespread adoption in adaptive radiotherapy can be justified.

### 3.2 The role of radiomics and artificial intelligence in prognostic assessment for cervical cancer radiotherapy

#### 3.2.1 Prediction of early treatment response in cervical cancer

Radiomics has shown potential for identifying patients who are likely to respond to or resist therapy before or early during treatment, thereby supporting adaptive radiotherapy strategies (58, 59). Gui et al. (60) developed an MRI-based radiomics model using a random forest algorithm in 183 patients with LACC, achieving an AUC of 0.80 for predicting pathological complete response. Liu et al. (61) reported that changes in the tumor-to-disc signal intensity ratio derived from T2-weighted imaging (T2WI) were independently associated with residual disease, with a threshold of 0.65 indicating elevated risk.

Zhang et al. (62) combined intravoxel incoherent motion parameters, including the perfusion fraction and diffusion coefficient, with 3D texture features to construct a predictive model for CCRT response. The model achieved AUC values of 0.987 in the training cohort and 0.984 in the validation cohort. However, such high discrimination performance in relatively small cohorts raises concerns regarding potential overfitting and warrants cautious interpretation.

Dynamic imaging biomarkers have also been investigated. Changes in the apparent diffusion coefficient (ADC) derived from diffusion-weighted imaging during the early treatment phase have been reported as potential early response indicators. Watanabe et al. (63) demonstrated that an insufficient increase in ADC values during CCRT, defined as less than 33%, was associated with a higher risk of disease recurrence. These early imaging-derived markers may facilitate timely treatment adaptation, including modification of radiation dose or target volumes during therapy.

In contrast, not all studies have demonstrated consistent predictive value. Collarino et al. (64) reported limited performance of a positron emission tomography (PET)-based radiomics model, potentially due to metabolic heterogeneity. Autorino et al. (65) developed a T2WI-based radiomics model for predicting pathological complete response, achieving an AUC of 0.80.

Despite promising discrimination performance, most early response models are based on retrospective single-center cohorts and lack independent external validation. Extremely high AUC values reported in small datasets warrant cautious interpretation due to potential overfitting. In addition, evidence is still lacking on whether early prediction of response meaningfully alters treatment strategy or improves patient outcomes.

#### 3.2.2 Prediction of survival outcome in cervical cancer

Conventional staging systems provide limited insight into intratumoral heterogeneity, which has stimulated increasing interest in radiomics-based survival modeling (66).

In a multicenter cohort of 582 patients, Lin et al. (67) reported that combining the radiomics score with motion-corrected imaging improved the prediction of 3-year OS, achieving an AUC of 0.862 and outperforming TNM staging. Shi et al. (68) integrated PET/CT-based radiomics features with inflammatory biomarkers using unsupervised bidirectional clustering, achieving a concordance index (C-index) of 0.691 and an AUC of 0.88 for OS prediction following CCRT.

However, not all investigations have demonstrated consistent predictive performance. Florit et al. (12)reported that early fluorodeoxyglucose PET-derived delta radiomics features did not reliably predict disease-free survival (DFS) in patients with LACC undergoing radical surgery after CCRT. Although some models showed marginal improvement over clinical models for OS prediction, the incremental benefit was limited. Wagner-Larsen et al. (69) further demonstrated that dynamic MRI-based radiomics changes during CCRT independently predicted progression-free survival (PFS), highlighting the value of longitudinal imaging biomarkers. Wang et al. (70) developed the CerviPro model by integrating clinical variables, conventional radiomics features, and DL architectures. Although the model achieved strong performance in training and internal validation cohorts, performance declined in external validation, underscoring challenges in generalizability and translational readiness. Future studies should emphasize longitudinal imaging integration, multimodal feature fusion, and optimization of functional imaging parameters to enhance robustness and clinical applicability.

Recent investigations have expanded beyond tumor-centric analysis. Park et al. (71) demonstrated that MRI-derived lymph node histogram features achieved superior DFS prediction compared with primary tumor features, with a C-index of 0.72 versus 0.69. Li et al. (72) incorporated spleen radiomics features and systemic inflammatory markers, reporting an AUC of 0.895 for PFS prediction when combined with FIGO stage, neutrophil-to-lymphocyte ratio, and spleen volume changes. These findings support the hypothesis that radiomic signatures reflecting the tumor microenvironment and systemic immune status provide complementary prognostic information.

While survival prediction represents a comparatively mature area of radiomics research in CC, methodological heterogeneity remains substantial (73). Many models rely on internal validation alone, and the incremental benefit over established clinical staging systems is sometimes modest. Whether radiomics-based risk stratification leads to measurable changes in treatment intensity or follow-up strategies has yet to be clearly demonstrated.

#### 3.2.3 Prediction of recurrence and metastasis risk in cervical cancer

The primary causes of treatment failure in CC are distant metastases and local recurrence (74). Early identification of high-risk patients enables more effective implementation of intensive adjuvant therapy and closer follow-up. Dankulchai et al. (75) developed a prediction model using pre-treatment T2WI radiomics features to predict local recurrence following CCRT. However, no radiomics markers were found to be significantly associated with OS.

Kawahara et al. (76) developed a multisequence MRI-based recurrence model that achieved an AUC of 0.94, demonstrating the value of integrating imaging features. Yusufaly et al. (77) incorporated non-tumor PET/CT radiomics features from muscle and fat to improve recurrence prediction, achieving a hazard ratio of 0.019 for risk stratification, which substantially outperformed stage-based models. Similarly, Mu et al. (78) employed PET/CT habitat radiomics to identify aggressive tumor subregions that predicted PFS and OS.

Recurrence prediction models suggest improved risk stratification compared with conventional staging; however, many are developed in relatively small cohorts and lack independent validation. Variability in imaging protocols and feature extraction strategies further complicates reproducibility across institutions. Moreover, the practical implications of identifying high-risk patients remain insufficiently defined in terms of adjuvant therapy selection or surveillance planning.

#### 3.2.4 Integrative assessment of prognostic modeling

In conclusion, studies on early response, survival, and recurrence prediction illustrate the expanding scope of radiomics in CC radiotherapy. Detailed performance metrics for survival modeling are summarized in **Table 1**, where many models demonstrate favorable discrimination. However, direct comparison across studies remains challenging because of heterogeneity in imaging protocols, feature preprocessing, modeling strategies, and outcome definitions.

Beyond performance metrics, **Table 4** provides an overview of methodological characteristics and translational readiness according to the Structured Translational Appraisal Framework. While several investigations incorporate internal resampling and, in some cases, multicenter cohorts, validation strategies remain inconsistent, and prospective evaluation is lacking. Importantly, statistical discrimination alone does not necessarily reflect clinical maturity.

Across domains, several cross-cutting challenges emerge. Harmonization of imaging acquisition and feature extraction remains limited, restricting reproducibility across institutions. The balance between model complexity and available sample size varies considerably, making it difficult to assess robustness with confidence. In addition, the connection between risk prediction and clearly defined clinical decision pathways is often insufficiently articulated.

Collectively, available evidence suggests that most prognostic models remain within exploratory or early validation phases of development. Further advancement will require more consistent validation practices and clearer articulation of how predictive information informs clinical decision-making.

### 3.3 Radiomics and artificial intelligence for predicting radiotherapy toxic side effects in cervical cancer

#### 3.3.1 Clinical challenges and predictive value of radiotherapy toxic side effects

Despite advances in CCRT and brachytherapy, treatment-related toxicities remain a major clinical challenge in LACC (79, 80). Acute and late adverse effects, including radiation enteritis, cystitis, and hematologic toxicity, are common and may compromise quality of life and treatment adherence. Substantial inter-individual variability in toxicity risk is observed even among patients receiving similar dose regimens. This heterogeneity underscores the limitations of conventional DVH-based prediction models, which rely primarily on summary dose metrics and do not account for individual biological susceptibility (81).

Radiomics and AI provide a data-driven strategy to capture subtle imaging phenotypes of normal tissues that may reflect intrinsic radiosensitivity. Accurate toxicity prediction before or early during treatment may support individualized dose optimization, improved OARs sparing, and proactive supportive care. Such approaches may enhance the therapeutic balance between treatment efficacy and safety.

#### 3.3.2 Radiomics-based prediction of gastrointestinal and genitourinary toxicity

Several studies have investigated radiation-induced proctitis and genitourinary toxicity. Zhang et al. (82) applied the SAM-Med2, a DL–based segmentation framework, to extract radiomics features from pre-treatment CT images obtained before CCRT. The model achieved an AUC of 0.73 for predicting radiation proctitis. This study suggested that DL-assisted segmentation may improve feature reproducibility.

Lucia et al. (83) combined DVH parameters and clinical variables within a normal tissue complication probability model. The integrated approach achieved AUC values of 0.83 for late rectal toxicity and 0.82 for genitourinary toxicity. These findings support the value of integrating dosimetric and radiomics information.

Xue et al. (84) developed a model based on MRI radiomics features extracted from images acquired before and after CCRT. The model achieved an AUC of 0.90 for predicting radiation-induced proctitis. After incorporating additional clinical variables, the AUC increased to 0.98. However, such near-perfect discrimination in a single-center cohort without independent external validation raises concerns regarding potential overfitting and limited generalizability. Wei et al. (85) constructed a CT-based model to predict severe radiation proctitis. After integrating clinical and dosimetric factors, the model achieved an AUC of 0.685.

Despite encouraging discrimination performance in certain cohorts, most toxicity prediction models remain retrospective and are seldom validated across independent institutions. The extent to which these models can reliably guide dose modification or prophylactic intervention strategies remains uncertain.

#### 3.3.3 Prediction of hematologic toxicity and bone marrow injury

Pelvic bone marrow toxicity has emerged as an important research focus in CC radiotherapy. Luo et al. (86) achieved an AUC of 0.927 by combining clinical factors with characteristics taken from pelvic bone marrow MRI images to create a predictive model for acute hematologic toxicity. Yue et al. (87) incorporated CT-derived bone marrow radiomics features, dose metrics, and clinical variables to predict acute hematologic toxicity. The model achieved an AUC of 0.802 in patients receiving CCRT and 0.737 in those treated with radiotherapy alone. These findings suggest that integrating dosimetric and radiomics features may improve early identification of bone marrow susceptibility.

Tang et al. (88) developed a composite model that combined dose-based scores with a clinical radiomics score to predict moderate to severe myelosuppression. The integrated model achieved an AUC of 0.743 and outperformed the conventional clinical and DVH model, which had an AUC of 0.650. Decision curve analysis demonstrated superior net benefit, indicating potential clinical utility.

Mechanistic imaging studies have further explored radiation-induced bone marrow injury. Wang et al. (89) used MRI with the iterative decomposition of water and fat with echo asymmetry and least squares estimation sequence to quantify pelvic bone marrow fat content. Bone marrow fat increased by an average of 58.5% after CCRT. The magnitude of fat increase was significantly associated with cumulative radiation dose. Fat changes within the 5 to 10 Gray dose region correlated with neutrophil and lymphocyte nadirs. These results suggest that even low-dose irradiation may substantially impair hematologic function.

Qin et al. (90) applied multi-sequence MRI radiomics to examine dose-dependent associations between first-order texture features and hematologic decline. The strongest associations were observed in low-dose irradiation regions. Collectively, these findings indicate that low-dose pelvic irradiation contributes to hematologic injury and should be carefully considered during treatment planning.

Although integration of radiomics and dosimetric features appears promising for identifying patients at risk of hematologic toxicity, validation strategies remain heterogeneous. Further work is needed to clarify how these predictive signals can be operationalized within treatment planning workflows.

#### 3.3.4 Methodological considerations and translational readiness in toxicity prediction

The predictive performance of radiomics-based toxicity models across gastrointestinal, genitourinary, and hematologic endpoints is summarized in **Table 2**. Several studies report favorable AUC values, particularly when radiomics features are combined with dosimetric and clinical variables. However, performance varies across toxicity subtypes and validation cohorts, reflecting differences in imaging modalities, endpoint definitions, and modeling strategies.

Beyond discrimination metrics, **Table 5** outlines the methodological characteristics and translational maturity of these investigations according to the Structured Translational Appraisal Framework. While a subset of studies incorporates relatively large cohorts or limited external validation, most remain retrospective and heterogeneous in validation design. In addition, reporting of calibration, reproducibility across imaging platforms, and integration within treatment planning workflows remains inconsistent.

Collectively, available evidence suggests that radiomics-based toxicity prediction remains largely within exploratory or early validation phases of development. Further advancement will depend not only on improved predictive stability but also on a clearer definition of how toxicity risk stratification informs dose optimization, prophylactic intervention, and supportive care strategies.

## 4 Discussion and future directions

**Figure 3:**
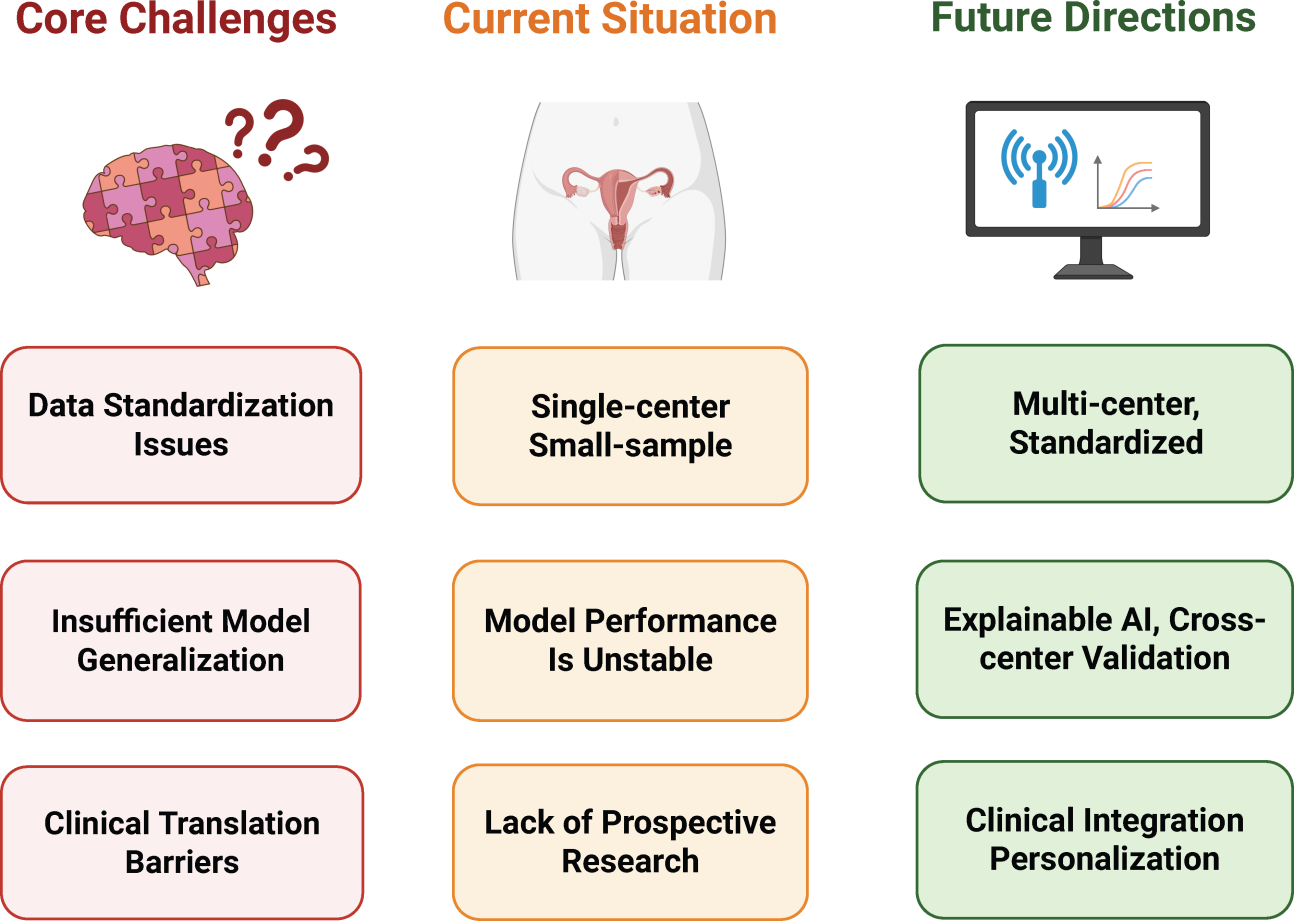
Core challenges and future directions.

As summarized in **Figure 3**, radiomics and AI in CC radiotherapy have achieved notable progress while still facing several key challenges and offering important future directions. Advances are most evident in automated segmentation, prognostic modeling, and toxicity prediction. However, when considered collectively, the current body of evidence remains methodologically heterogeneous and largely exploratory in terms of clinical translation (91).

### 4.1 Methodological maturity and statistical robustness

Radiomics research in CC radiotherapy has achieved notable technical advancement across segmentation, prognostic modeling, and toxicity prediction, although methodological maturation remains ongoing.

Much of the literature relies on retrospective designs and relatively small cohorts in relation to the dimensionality of extracted radiomic features. In survival and toxicity prediction research, high-dimensional feature sets are frequently modeled despite a limited number of outcome events, increasing susceptibility to overfitting. Although regularization and internal resampling are commonly employed to improve model stability, independent external validation across heterogeneous institutions and imaging platforms remains limited.

Performance evaluation is largely centered on discrimination metrics such as AUC, C-index, or DSC While informative, these measures capture only one aspect of methodological quality. Calibration, robustness across scanners and acquisition protocols, reproducibility of segmentation workflows, and harmonization strategies are inconsistently reported, leaving generalizability uncertain (92).

Beyond model-level performance considerations, substantial heterogeneity in imaging protocols, preprocessing pipelines, feature extraction procedures, and validation strategies further limits cross-study comparability. Even in multicenter settings, similarities in imaging equipment may constrain the true assessment of external validity (93).

In light of emerging AI reporting and validation standards, most published models remain at exploratory or early validation stages. Evaluation in routine clinical settings remains limited, indicating that the field reflects technical feasibility more than full methodological readiness.

### 4.2 From predictive accuracy to clinical decision impact

Improvement in discrimination metrics does not in itself justify clinical implementation. In addition to methodological robustness, clinical translation remains a major challenge. Although high AUC or C-index values have been reported, the clinical significance of these models depends on their ability to inform decision-making and improve outcomes.

Radiomics models are often proposed to support adaptive radiotherapy, individualized dose modulation, intensified systemic treatment, or personalized surveillance. Although conceptually aligned with precision oncology, empirical evidence showing that model-informed strategies alter management or improve patient-centered outcomes remains limited.

Even when statistical performance appears strong, models may offer limited practical value if they fail to influence management decisions beyond existing clinical criteria. Successful integration into clinical workflows also depends on practical considerations such as inference time, interpretability, regulatory requirements, and institutional acceptance, factors that are rarely addressed in current reports.

Prospective trials demonstrating patient-level benefit and decision impact are still lacking; future studies could be designed to test whether radiomics-informed strategies influence therapeutic choices and lead to measurable clinical benefit.

### 4.3 Practical barriers to implementation

Practical challenges related to implementation remain underreported. Few studies address integration with treatment planning systems, inference time within routine clinical workflows, regulatory considerations, or data governance requirements. Model interpretability and clinician trust are additional determinants of adoption, especially for complex DL architectures. Economic evaluation is rarely reported, leaving cost-effectiveness and scalability uncertain. Even statistically robust models may encounter implementation failure if performance monitoring, recalibration mechanisms, and quality assurance procedures are not embedded into routine clinical systems. Translational maturity depends on statistical performance as well as operational feasibility and institutional acceptance.

### 4.4 Future directions

Future progress will depend on multicenter validation under harmonized imaging standards. Reporting would benefit from alignment with emerging methodological guidelines for AI in medical imaging. Calibration analysis and clinical utility assessment are likely to become increasingly important components of model evaluation.

Most importantly, future investigations will need to clarify whether radiomics-informed strategies meaningfully change management and improve patient outcomes. The long-term value of radiomics in CC radiotherapy depends on demonstrable patient benefit and sustainable integration into clinical workflows. In the absence of such evidence, these technologies remain promising but investigational.

## 5 Conclusion

Radiomics and AI offer promising avenues to advance precision radiotherapy in CC across segmentation, prognostic modeling, and toxicity prediction. However, current evidence remains largely exploratory, and improved statistical performance does not yet translate into demonstrated clinical benefit. Rigorous prospective validation and decision-impact studies are required before these approaches can be integrated into routine practice.
